# Assessing Cognitive behavioural Therapy in Irritable Bowel (ACTIB): protocol for a randomised controlled trial of clinical-effectiveness and cost-effectiveness of therapist delivered cognitive behavioural therapy and web-based self-management in irritable bowel syndrome in adults

**DOI:** 10.1136/bmjopen-2015-008622

**Published:** 2015-07-15

**Authors:** Hazel Everitt, Sabine Landau, Paul Little, Felicity L Bishop, Paul McCrone, Gilly O'Reilly, Nicholas Coleman, Robert Logan, Trudie Chalder, Rona Moss-Morris

**Affiliations:** 1 Primary Care and Population Sciences, Faculty of Medicine, University of Southampton, Southampton, UK; 2 Department of Biostatistics, Institute of Psychiatry, Psychology and Neurosciences, Psychology and Neuroscience Kings College, London, UK; 3 Centre for Applications of Health Psychology, University of Southampton, Southampton, UK; 4 Institute of Psychiatry, Psychology and Neuroscience, Kings College, London, UK; 5 Department of Gastroenterology, Southampton University Hospital, Southampton, UK; 6 Department of Gastroenterology, Kings College Hospital, London, UK; 7 Academic Department of Psychological Medicine, Kings College, London, UK; 8 Health Psychology Section, Institute of Psychiatry, Psychology and Neuroscience, Kings College, London, UK

**Keywords:** PRIMARY CARE

## Abstract

**Introduction:**

Irritable bowel syndrome (IBS) affects 10–22% of the UK population, with England's annual National Health Service (NHS) costs amounting to more than £200 million. Abdominal pain, bloating and altered bowel habit affect quality of life, social functioning and time off work. Current treatment relies on a positive diagnosis, reassurance, lifestyle advice and drug therapies, but many people suffer ongoing symptoms. Cognitive behaviour therapy (CBT) and self-management can be helpful, but availability is limited.

**Methods and analysis:**

To determine the clinical- and cost-effectiveness of therapist delivered cognitive behavioural therapy (TCBT) and web-based CBT self-management (WBCBT) in IBS, 495 participants with refractory IBS will be randomised to TCBT plus treatment as usual (TAU); WBCBT plus TAU; or TAU alone. The two CBT programmes have similar content. However, TCBT consists of six, 60 min telephone CBT sessions with a therapist over 9 weeks, at home, and two ‘booster’ 1 hour follow-up phone calls at 4 and 8 months (8 h therapist contact time). WBCBT consists of access to a previously developed and piloted WBCBT management programme (Regul8) and three 30 min therapist telephone sessions over 9 weeks, at home, and two ‘booster’ 30 min follow-up phone calls at 4 and 8 months (2½ h therapist contact time). Clinical effectiveness will be assessed by examining the difference between arms in the IBS Symptom Severity Score (IBS SSS) and Work and Social Adjustment Scale (WASAS) at 12 months from randomisation. Cost-effectiveness will combine measures of resource use with the IBS SSS at 12 months and quality-adjusted life years.

**Ethics and dissemination:**

This trial has full ethical approval. It will be disseminated via peer reviewed publications and conference presentations. The results will enable clinicians, patients and health service planners to make informed decisions regarding the management of IBS with CBT.

**Trial registration number:**

ISRCTN44427879.

**Strengths and limitations of this study**

- To date, Assessing Cognitive behavioural Therapy in Irritable Bowel (ACTIB), when completed, will be the largest trial, worldwide, to address the clinical-effectiveness and cost-effectiveness of cognitive behaviour therapy (CBT) for irritable bowel syndrome (IBS) and has the advantage of comparing a low intensity web-based CBT (WBCBT) with a higher intensity telephone therapist delivered CBT (TCBT).
- ACTIB will recruit from both primary and secondary care, inviting a broad range of patients with refractory IBS from specialist as well as community settings. This will aid generalisability of the findings.
- Owing to the online nature of the Low intensity CBT arm, patients without internet access will be unable to participate. However, internet access in the UK is currently over 75% and those without home access could use public computers (eg, local library).
- Participants aged over 60 years must have had a consultant review to exclude other serious causes of their bowel symptoms in the past 2 years, because colorectal cancer is more common in those over 60 years of age and guidelines recommend that changes in bowel habit in this group require hospital tests beyond the scope of this trial.

## Introduction

IBS is a common chronic gastrointestinal disorder that affects 10–22% of the UK population and costs the National Health Service (NHS) in England over 200 million pounds a year.^1^ ^2^ Abdominal pain, bloating and altered bowel habit affect quality of life, social functioning and time off work.^3^ ^4^ Treatment commonly relies on a positive diagnosis, reassurance, lifestyle advice and drug therapies. However, the evidence base for commonly used medications such as bulking agents and antispasmodics is limited,^4^ and many patients suffer ongoing symptoms.

Face to face cognitive behavioural therapy (CBT) has been shown to be helpful for IBS, reducing IBS symptom severity and improving QOL measures,^5–7^ and it has the potential to have long-term benefits, whereas medications are aimed at symptom relief. However, CBT is not currently routinely offered to patients with IBS due to poor availability. Additionally, face to face CBT in this setting was not shown to be cost effective in a Cochrane review^5^ and there are problems with limited concordance^5^ with face to face therapy. For instance, in the Kennedy trial,^6^ fewer than half of the participants were considered to have completed therapy by the end of the intervention and 41% were recorded as declining therapy or dropping out, often due to time issues such as work and child care commitments. Nevertheless, National Institute for Health and Care Excellence (NICE) guidance^4^ recommends CBT for patients with refractory IBS symptoms (ie, ongoing symptoms after 12 months despite being offered appropriate medications and lifestyle advice).

A potential approach is to consider web-based CBT for IBS. Web-based CBT has been shown to be helpful for a number of long-term conditions, for example, depression,^8^ tinnitus^9^ and fatigue in multiple sclerosis,^10^ and is recommended in Guidelines for the management of depression.^11^ It could be a cost-effective way of providing help to those with IBS. Recent small pilot trials show promise for web-based CBT in IBS,^12–14^ but indicate that some therapist input is needed. Web-based delivery has the advantage that it can be accessed at a time and place convenient to the participant, can be undertaken at a pace that suits each individual’s circumstances, and does not require extra travel time and costs. The increasing availability of the Internet makes this a good medium to provide easily accessible patient information and self-management programmes. The majority of households in the UK now have web access. This is, therefore, an ideal time to assess and disseminate new web-based interventions. Members of this team have already developed a CBT website to support patients with IBS (Regul8) and trialled it among 135 patients with more than 90% follow-up in the National Institute of Health Research (NIHR)-funded MIBS study.^14^ Even with this (underpowered) sample, and very minimal nurse input, Subjects Global Assessment of Relief (SGA) scores (ie, relief from IBS symptoms) and their Enablement Scores (sense of control over their IBS) were significantly improved in the Regul8 groups compared to the non-website group at 3-month follow-up.

This trial comparing the clinical-effectiveness and cost-effectiveness of therapist delivered CBT (TCBT) and a lower intensity therapist supported web-based self-management programme will provide evidence to enable clinicians, patients and health service planners to make informed decisions regarding the management of IBS with CBT.

## Main Research Question

What is the clinical-effectiveness and cost-effectiveness of TCBT and web-based CBT (WBCBT) for patients with refractory irritable bowel syndrome?

## Research objectives

*Primary objectives*

- 1. Estimate the clinical effectiveness of therapist delivered telephone CBT (TCBT) plus treatment as usual (TAU) for reducing the severity and impact of IBS symptoms compared to TAU alone at 12 months after randomisation.
- 2. Estimate the clinical effectiveness of Regul8, a previously developed web based CBT programme, with minimal therapist support (WBCBT) plus TAU for reducing the severity and impact of IBS symptoms compared to TAU alone at 12 months after randomisation.

*Secondary objectives*

- 3. To compare the cost-effectiveness of TCBT and WBCBT in comparison to TAU over the 12-month follow-up period.
- 4. To estimate (1) and (2) at 3 and 6 months after randomisation.
- 5. To assess whether TCBT and/or WBCBT have a positive impact on relief of IBS symptoms, quality of life, enablement, anxiety and depression compared to TAU at 3, 6 and 12 months follow-up, and acceptability of the treatment.

*Tertiary aims*

- 6. To investigate possible cognitive and behavioural mediators or processes of clinical improvement for both the TCBT and WBCBT.
- 7. To examine predictors and moderators of outcome.

## Methods and analysis

### Design

Three arm multicentre randomised controlled trial.

### Method

Four hundred and ninety-five patients with refractory IBS will be individually randomised to TCBT+TAU, or WBCBT (a previously developed self-management CBT website with low levels of therapist support)+TAU, or TAU alone for 9 weeks with 12-month follow-up.

### Setting

Treatment will take place at participants’ homes via telephone and internet. Therapists will be based at the South London and Maudsley NHS Foundation Trust (SLAM). Participants will be recruited from London and the South Coast of England from primary and secondary care.

### Target population

*Inclusion Criteria*: Adults (18 years and over) with refractory IBS. Refractory IBS is defined for this study as fulfilling the ROME III criteria for IBS^15^ and reporting ongoing clinically significant symptoms determined by a IBS symptom severity score (IBS-SSS) of 75 or more. Patients need to have been offered first-line therapies (eg, antispasmodics, antidepressants or fibre based medications) and have continuing IBS symptoms for 12 months or more. Potential participants aged over 60 years will only be included if they have had a consultant review in the previous 2 years to confirm that their symptoms are related to IBS and that other serious bowel conditions have been excluded. This is because there is an increased risk of bowel cancer in the over 60 years’ age group and clinical guidance suggests further investigations should be undertaken in this group.^4^

*Exclusion criteria*: Unexplained rectal bleeding or weight loss, diagnosis of inflammatory bowel disease, coeliac disease, peptic ulcer disease or colorectal carcinoma. People unable to participate in CBT due to speech or language difficulties or those with no access to an internet computer to be able to undertake the WBCBT or who have received CBT for IBS in the past 2 years, and those who have had previous access to the Regul8 website or who are currently participating in an IBS/intervention trial.

*Withdrawal criteria*: Participants will be withdrawn from the trial if there are any concerns regarding informed consent. Participants can also withdraw if they choose, without giving a reason. If a participant withdraws consent for research follow-up during the trial, the trial team will be informed. Information will be collected in the Drop-out Report Form and, where possible, reason for drop out will be recorded.

### Planned interventions

Two methods of delivering CBT are being assessed in this study: TCBT and a lower intensity WBCBT—the Regul8 website with some therapist support.

There are three key differences between the therapy trial arms

1. The use of a CBT self-management patient manual in the TCBT arm versus access to Regul8, an interactive, tailored CBT self-management website^14^ for the WBCBT arm.
2. The amount of therapist contact time/intensity of the intervention—TCBT participants will receive a total of 8 h of telephone therapy contact time compared to 2.5 h in WBCBT.
3. The TCBT telephone sessions will be formulation driven and, although based on the content of the sessions/chapters of the patient manual detailed below, order and extent to which these are covered will be individualised. For WBCBT, patients are encouraged to work sequentially through the Regul8 sessions, although the therapist may suggest they focus more on some sessions than on others.

The CBT content of the two treatments is the same and is based on an empirical cognitive behavioural model of IBS.^16^ The model specifies that factors such as stress and/or gastric infection trigger the symptoms of IBS, which are then maintained by patients’ cognitive, behavioural and emotional responses to the symptoms. For instance, if a patient becomes anxious (emotion) about the symptoms, believes he/she has no control over them (cognitions) and responds by avoiding social situations (behaviour), this can increase anxiety and maintain symptoms through the link between a heightened autonomic nervous system and the enteric nervous system. This model was used to structure the content of the therapy sessions in our Regul8 website for the MIBS pilot study,^14^ which, in turn, drew from two efficacious IBS RCTs conducted by members of our research team, a nurse-delivered CBT trial^6^ and a trial of a more minimal CBT based self-management programme.^16^ The therapy consists of education, behavioural and cognitive techniques, aimed at improving bowel habits, developing stable, healthy eating patterns, addressing unhelpful thoughts, managing stress, reducing symptom focusing and preventing relapse. A summary of the sessions and related homework tasks are presented in table 1.

**Table 1:** Summary of the Self-Management Sessions included in the Regul8 website and the TCBT patient manual

**Session 1:** Understanding your IBS	Rationale for self-management, which includes the following explanations 1. Possible causes of IBS and illustrative physiology of the digestive system together with the functional changes that occur in the gut as a result of IBS 2. How the autonomic nervous system (‘fight-or-flight’ stress system) may interact with the enteric nervous system
**Session 2:** Assessing your symptoms	Self-assessment of the interaction between thoughts, feeling and behaviours, and how these can impact on stress levels and gut symptoms Development of a personal model of IBS that incorporates these elements Homework: Daily diaries of the severity and experience of IBS symptoms, in conjunction with stress levels and eating routines/behaviours
**Session 3:** Managing symptoms and eating	Review of the symptom diary Behavioural management of the symptoms of diarrhoea and constipation, and common myths in this area, are discussed. Goal setting is explained The importance of healthy, regular eating and not being overly focused on elimination is covered Homework: Goal setting for managing symptoms and regular/healthy eating. Goal setting, monitoring and evaluation continue weekly throughout the programme
**Session 4:** Exercise and activity	Importance of exercise in symptom management is covered Identifying activity patterns such as resting too much in response to symptoms or an all-or-nothing style of activity is addressed. Homework: Goal setting for regular exercise and managing unhelpful activity patterns if relevant
**Session 5:** Identifying your thought patterns	Identifying unhelpful thought (negative automatic thoughts) in relation to high personal expectations and IBS symptoms is introduced Link between these thoughts, feelings, behaviours and symptoms is reinforced Homework: Goal setting plus daily thought records of unhelpful thoughts related to personal expectations and patterns of over activity
**Session 6:** Alternative thoughts	The steps for coming up with alternatives to unhelpful thoughts are covered together with personal examples Homework: Goal setting plus daily thought records including coming up with realistic alternative thoughts
**Session 7:** Learning to relax, improving sleep, managing stress and emotions	Basic stress management and sleep hygiene are discussed Diaphragmatic breathing, progressive muscle relaxation and guided imagery relaxation, are presented in video and audio formats Identifying common positive and negative emotions, and the participant's current ways of dealing with these New strategies to facilitate expression of emotion as well as coping with negative or difficult emotions are discussed Homework: Goal setting for stress management, good sleep habits and emotional processing
**Session 8:** Managing flare-ups and the future	The probability of flare-ups is discussed and patients are encouraged to develop achievable, long-term goals, and to continue to employ the skills they have learnt throughout the manual to manage flare-ups and ongoing symptoms

IBS, Irritable bowel syndrome; TCBT, therapist delivered cognitive behavioural therapy.

Participants randomised to TCBT will be contacted by one of the therapist teams to organise the therapist telephone sessions, and will be sent a detailed CBT manual including homework sessions to support the sessions. The TCBT arm will have six 1 h telephone sessions with a CBT therapist over 9 weeks as well as homework tasks. They will also receive two 1 h booster sessions at 4 and 8 months. CBT will be delivered by telephone rather than face to face, as both have similar efficacy,^17^ improve accessibility, are efficient and less costly, and can be readily delivered from specialist-centralised services across a large geographic area.^17–19^

Participants randomised to the WBCBT arm will be provided with log-in access to Regul8. They will be advised to start working through the eight online weekly sessions and homework tasks, and will receive weekly automated email reminders. In addition, they will receive three brief 30 min telephone therapy support calls over 9 weeks and two 30 min booster sessions at 4 and 8 months. The telephone CBT sessions for the WBCBT arm are undertaken while they are working through the website self-management programme to help engagement with the CBT programme. Participants will also be able to email the therapist regarding queries about the website programme, during the study. Limited therapist input has been included in this condition as several small trials of web-based^12^ ^13^ or manual-based^16^ CBT for IBS have shown promising results but indicated that therapist input is important to maintain participant engagement. Qualitative interviews with participants from the MIBS^20^ study also highlighted the benefit of the telephone support session in improving patient understanding of Regul8.

In both therapy arms, medical questions will not be addressed by the therapists, and participants will be advised to seek medical advice if they have medical queries. Booster sessions are included in both arms to discuss any setbacks and to reinforce positive symptom management.

Secure website pass-wording will ensure non-contamination of treatments. Patients in the TCBT arm will also be requested not to share their manual with others.

### Therapists

CBT trained therapists (clinical psychologists or cognitive behavioural nurse therapists) will provide the telephone CBT sessions for the TCBT as well as the WBCBT arms of the study. Each therapist will receive training in both therapy protocols. Competency ratings will be assessed for the first two patients in each active treatment arm using a modified version of the developed rating scale that had previously been used for assessing competency in delivering CBT for fatigue in primary care.^21^

### Therapy manuals

A therapy manual has been written and will be used as the basis to train the therapists. It consists of: information and procedures about the trial, background information about IBS, a description of the anatomy and physiology of the bowel, a cognitive behavioural model of IBS, aspects of the therapeutic alliance, sections on ways of engaging the patient and on various cognitive, behavioural and emotional strategies, information on how to utilise supervision and how to overcome difficulties in the treatment process, and a description of the two different approaches, TCBT and WBCBT. The manual also includes protocols for the five telephone support sessions for the Regul8 website, including instructions for the optimum setting for the telephone calls, in other words, a quiet environment without interruptions and keeping prompt-sheets handy for the sessions so that the therapist can check that all the key points are covered.

### Therapy training

Therapists will receive two days of training. Prior to the training days, they will be asked to explore the Regul8 website and read the patient manual. Training consists of information about IBS including diagnosis, aetiology and evidence based practice. The IBS CBT treatment model is presented alongside explanations of how the bowel works and how this relates to functional disturbances such as changes in motility and sensitivity in the gut in IBS. Therapists are taught to include predisposing, precipitating and perpetuating factors in their assessment and to use these in a shared conceptualisation with the patient. Obstacles to engagement and strategies for dealing with these are discussed. The specifics of each of the sessions in table 1 are then covered. Finally, therapists receive an overview of trial protocol including recording the timing and length of sessions, any deviations from protocol including sessions missed or drop out, and confidential storage of audiorecordings.

### Therapy supervision

Post-training, therapists will receive monthly 1.5 h group supervision with TC, who covers the TCBT, and RMM, who covers the WBCBT. TC and RMM listen to one audiorecording from each therapist, and rate these recordings using the therapist rating scale, prior to supervision.^21^ These sessions will be discussed with the group in supervision. Therapists will also have the opportunity to discuss any problem areas or challenging patients. Regular supervision will ensure that the therapists adhere to the protocols in each arm and that the quality of the therapy is maintained.

### Treatment fidelity

All telephone therapy sessions will be audiorecorded for the purpose of assessing treatment fidelity. These recordings will be used for supervision during the study and to check fidelity throughout the trial. Supervisers will listen to one TCBT and one WBCBT tape per therapist per month. A subset of the audiorecordings will be analysed by two independent clinicians once the trial has ended. At least two sessions for every therapist (when available) and for therapy type will be rated in terms of adherence to the manual or web-based approach (5 item, 7-point Likert scale). The therapeutic alliance between therapist and participant will also be rated on a seven-point Likert scale used in a previous large RCT of treatments for chronic fatigue syndrome.^22^

### Treatment as usual

Patients in all three arms will receive TAU, with the control arm being TAU alone. TAU is defined as continuation of current medications, and usual general practitioner (GP) or consultant follow-up with no psychological therapy for IBS. All GPs or consultants involved in the study will receive a copy of the NICE Guidance for IBS at the start of the study, to ensure all clinicians have standard best practice information on IBS management. They will also receive a Desktop prompt to remind them of the guidelines and inclusion criteria. All participants will receive a standard information sheet on Lifestyle and Diet in IBS, based on the NICE guidance. Information will be collected on any changes in IBS treatments/management during the study, and numbers of GP and consultant consultations will be recorded for all three arms.

The TAU-alone participants will have access to the WBCBT website at the end of the trial follow-up period, but without the therapist support.

### Recruitment

Patients will be recruited from secondary and primary care.

We plan to recruit 495 participants over 22 months (23 randomised/month) from GP surgeries in two regions (Southampton and London) and Secondary Care Gastroenterology Clinics in two regions (Southampton and London (GSTT, King's College Hospital)).

Primary care patients will be identified by searching GPs’ lists for those with a diagnosis of IBS and by opportunistic recruitment of patients presenting with symptoms consistent with IBS. We will utilise the Clinical Research Network (CRN) to aid recruitment and retention of GP practices. We will include practices with urban and rural settings, and with a range of sociodemographic characteristics. GP practices willing to participate in the study will search their list for adult patients aged 18 years and above with a diagnosis of IBS. Potential participants will be contacted by letter (sent by the GP surgery) informing them about the trial and inviting them to take part. The GPs will check the lists of patients to be contacted prior to the invite letters being sent out to ensure that it is appropriate to contact them. The mailing will include the Assessing Cognitive behavioural Therapy in Irritable Bowel (ACTIB) patient information sheet. Participants who are interested in participating in the study will return a reply slip with their contact details in a prepaid response envelope to the research team. GPs will also be able to opportunistically provide information about the trial to potential recruits during their GP surgeries. Thus, if a patient with IBS attends a GP consultation, GPs will give them the patient information sheet regarding the trial, and the reply slip and envelope. Invite letters will be sent out from the identified GP practices in a stepwise manner over time, and response rates will be monitored to ensure adequate recruitment levels and a steady workload for the therapists.

Secondary care patients will be identified from gastroenterology (GI) clinics. Where available, clinic lists will be searched for patients with a diagnosis of IBS. Potential participants will be contacted by letter (sent from the clinic) informing them about the trial and inviting them to take part. The Consultants will check the lists of patients to be contacted prior to the invite letters being sent out to ensure that it is appropriate to contact them. The mailing will be as for the primary care patients. The consultants will also be able to opportunistically provide information about the trial to potential recruits during their clinics.

Adverts will also be placed in relevant GP and GI clinics and on NHS websites. Clinics and GP practices will have information packs to hand out to potential participants.

### Study procedures

**Figure 1:**
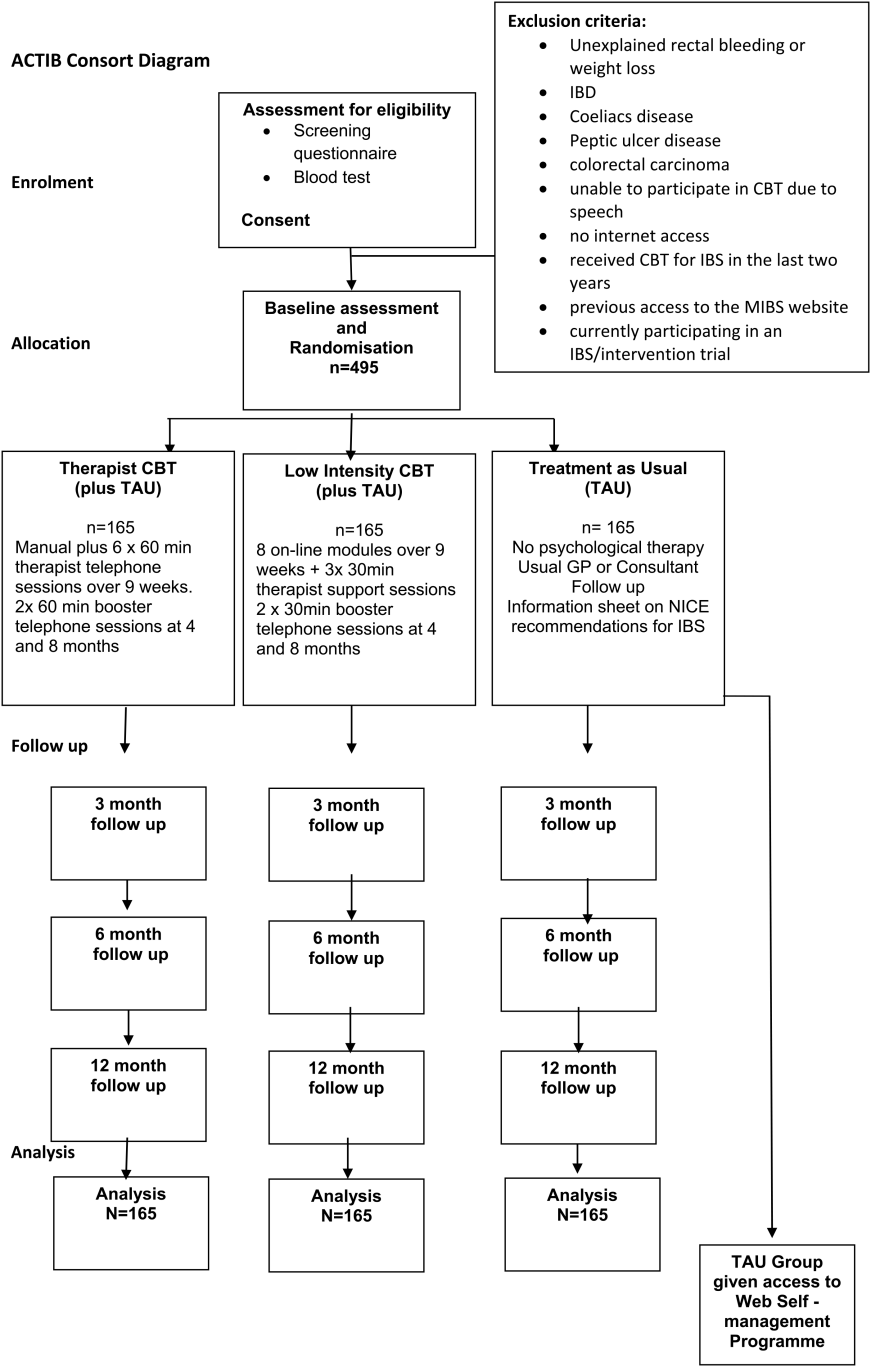
Consort diagram for Assessing Cognitive behavioural Therapy in Irritable Bowel (ACTIB).

Information is also in the Consort diagram (figure 1) and table 2 (screening and data collection).

**Table 2:** Screening and data collection across the trial: summary of the key trial processes from receipt of the invite reply from the potential participant to the data collection time points

CRF	Completed by	Database	Preconsent	Baseline	3 m	6 m	12 m	Ongoing or during treatment	Ref
Invite reply	P	RT	X						na
Screening Questionnaire	P/TT	M	X						na
Consent	P	R		X					na
Sample requisition form	RN	RT		X					na
Adverse events form	TT	M						X	na
Drop-out event form	TT	M						X	na
Note review form	TT	M					X		na
IBS-SSS	P	R		X	X	X	X		23
WASAS	P	R		X	X	X	X		24
SGA	P	R			X	X	X		25
EQ5D	P	R		X	X	X	X		26
Patient enablement	P	R			X	X	X		27
Hospital Anxiety and Depression Scale	P	R		X	X	X	X		28
Client Service Receipt Inventory	P	R		X	X	X	X		29
Cognitive Scale CG-FBD	P	R		X	X	X	X		30
B-IPQ for IBS	P	R		X	X	X	X		31
IBS Behavioural Responses Questionnaire	P	R		X	X	X	X		32
BES	P	R		X	X	X	X		33
“Impoverished Emotional Experience (IEE)” factor of the Emotional Processing Scale-25	P	R		X	X	X	X		34
PANAS	P	R		X	X	X	X		35
Demographics	P	R		X					na
About your IBS	P	R		X					na
Safety questions	P	R			X	X	X		na
Rating of satisfaction	P	R			X	X	X		22
Thoughts on my treatment	P	R			X	X	X		36
Therapist database	T	MT						X	na

BES, Beliefs about Emotions Scale; B-IPQ, Brief Illness Perception Questionnaire; CG-FBD, Cognitive Scale for Functional Bowel Disoders; CRF, Case Report Form; IBS, Irritable bowel syndrome; IBS-SSS, IBS Symptom Severity Score; M, MACRO Clinical Trials Unit database; MT, MACRO Therapist database; na, not significant; P, patient; PANAS, Positive and Negative Affect Schedule; SGA, Subject's Global Assessment of Relief; R, LifeGuide Regul8; RN, Research Nurse/Phlebotomist; RT, Research Team database; T, therapist; TT, trial team; WASAS, Work & Social Adjustment Scale.

Those responding to the recruitment invitation letter will be contacted by the study team to complete a screening process consisting of the Rome III criteria, and questions about exclusion and inclusion criteria to check if they fulfil the eligibility criteria for the study. They will be identified with a unique ID number. Any patient indicating they may have a ‘red flag’ symptom that would indicate the need for further investigations (ie, unexplained weight loss or rectal bleeding) will be referred back to their GP for further assessment and will not enter the study unless the GP feels the symptoms have been fully assessed and that the patient is suitable for study entry.

Those fulfilling the screening entry requirements will be contacted by one of the research team to make sure they are fully informed of trial procedures, and they will be sent the login and access details for the website in order for them to complete an on-line consent form. They will then be sent arrangement details to have a blood test for full blood count, tissue transglutaminase antibodies and C reactive protein (CRP), to exclude alternative diagnoses, such as anaemia that requires further investigation and coeliacs disease (as recommended for IBS diagnosis in the NICE guidelines^4^). The blood tests will be undertaken by practice nurses/GPs within the GP surgeries or by phlebotomists/research nurses at the secondary care sites. Samples will be sent to University Southampton Hospital pathology laboratory for testing and will then be destroyed. The results will be made available to the participants’ GP. If the blood tests are within normal limits, the participant can complete the baseline measures and can then be randomised. If the blood tests show an abnormal result, that is, a CRP over the normal laboratory range, anaemia or a positive test for coeliacs disease, the patient will not be randomised to the trial but will be referred back to his or her GP for further assessment.

### Randomisation

Randomisation will be provided by an independent randomisation service at the UKCRC registered King’s Clinical Trials Unit (CTU) and accessed by study sites via a web-based system. Randomisation will be at the level of the individual, using block randomisation with randomly varying block sizes, stratified by centre (Southampton GP practices, Southampton secondary care, London GP practices, London secondary care). Confirmation emails will be generated automatically and sent to relevant study site and coordination staff.

### Blinding

It is not possible in therapy trials to blind participants or therapists to treatment allocation, however, no hypotheses have been proposed as to the superiority of one treatment over the other. As the research assistants are responsible for allocating patients to therapists who have current availability, they will also be unblinded, however, the principal investigators and statisticians will remain blinded. All outcomes are patient reported and collected via the internet, following automated email reminders. The trial team member who will contact participants to capture primary outcome data by telephone on the short questionnaire for those who have not completed follow-up questionnaires after email reminders, will be blinded to the participants treatment group, to avoid bias.

Locked codes will be used for treatment allocation and the trial statisticians will be blinded to treatment allocation, as will be the Data Monitoring and Ethics Committee (DMEC), in order to take actions on the basis of the unblinded data alone. The majority of the data will be analysed blind and the codes only unlocked when necessary, to enable analysis of therapist effects.

### Data collection

Research data will be entered onto GCP compliant online data entry systems at CTU (InferMed MACRO) and Regul8 on the LifeGuide Platform. Invite reply data will be entered into the Research Team databases locally. Participant screening data will be collected by telephone and entered into MACRO by the study site staff. Baseline data will be collected prior to randomisation. Baseline and outcome data will be patient self-completed on a separate data collection section of the Regul8 website (as was carried out successfully for the MIBS study), away from the study team, thus avoiding any influence of the study team on the responses and reducing bias. This website will be maintained by the computer support team at Southampton, which is hosting the website. Participants will be given a unique password to log onto the website. Their data will be identified by a unique identification number and will be kept separate from any personal identifying data, to maintain confidentiality. A Therapist database on the CTU MACRO system will be completed by the therapists. It will record which therapist provided the sessions, number of sessions and other contact telephone calls, any drop outs from therapy and a therapist-recorded rating of patient change, adherence and acceptance of therapy model.

### Baseline measures

The screening questionnaire will capture baseline data including Rome III questionnaire, duration of IBS, type, previous CBT, medications previously taken for IBS, and inclusion and exclusion criteria. The participant will complete an online baseline assessment questionnaire, which includes the outcome measures detailed below plus sociodemographic details, current medication, medical history and medications, duration of IBS symptoms and previous or current psychiatric diagnoses.

### Outcome measures

Outcome data and questionnaires will be completed at baseline, 3, 6 and 12 months after randomisation, by all participants. Participants will be sent a reminder email at 3, 6 and 12 months, to prompt them to complete entering the data 1 week prior to the questionnaire due date. If it has not been completed within 1 week of the reminder, a further two reminders will be sent. One week after that, if no data have been entered, the research team will ring the participant to ask if they can collect the data by hard copy or over the telephone. 90% follow-up was achieved (at 12 weeks) by this method in the MIBS trial, which collected very similar baseline and outcome measures to those proposed for this study.

### Primary outcomes: IBS-SSS and WASAS

IBS Symptom Severity Score (IBS SSS)^23^ is widely used in IBS studies. It is a five item self-administered questionnaire measuring: severity of abdominal pain, duration of abdominal pain, abdominal distension/tightness, bowel habit and quality of life. (Maximum score 500: <75 normal bowel function, 75–174 mild IBS, 175–299 moderate IBS, 300–500 severe IBS). A 50 point change from baseline is regarded as clinically significant.^23^

The Work and Social Adjustment Scale (WASAS) measures the effect of the IBS on people's ability to work and manage at home, participate in social and private leisure activities, and maintain relationships.^24^ WASAS has been shown to be sensitive to change in IBS trials.^6^ ^16^ It has five aspects scored 0 (not affected) to eight (severely affected), total possible score 40.

### Secondary outcome measures

Two of the secondary outcomes will not be completed at baseline, only at follow-ups These include the SGA (Subject's Global Assessment of Relief)^25^ and the Patient Enablement Questionnaire.^16^

The SGA is frequently used in treatment trials to identify IBS responders to therapy.^25^ Participants rate their relief from IBS symptoms on a scale of 1–5 ranging from ‘completely relieved’ to ‘worse’. Scores are dichotomised so that patients scoring from 1 to 3 are considered responders and those scoring 4–5, non-responders.

The Patient Enablement Questionnaire^27^ assesses change in the participants’ ability to cope with their illness and life after treatment.

Mood will be measured by the Hospital Anxiety and Depression Scale (HADS),^28^ a well validated, commonly used, self-report instrument for detecting depression and anxiety in patients with medical illnesses.

The acceptability of the self-management treatment will be assessed using questions where patients rate the overall effectiveness of the programme, the efficacy of programme compared to other treatments they have tried and whether they enjoyed the programme.

The Client Service Receipt Inventory (CSRI)^29^ and EQ-5D^26^ will be used to gather information on use of health services, and health-related quality of life, respectively. The CSRI has been adapted for use in many economic evaluations and has been used in previous IBS evaluations. The EQ-5D is the most frequently used tool for generating quality-adjusted life years (QALYs), which are favoured by NICE.

### Adherence to therapy

Patients’ adherence to the treatments will be measured through recording the number of phone sessions and an automated count of web sessions accessed. Completing four or more sessions of the website and one or more of the telephone support calls will be deemed as compliant with the website. In the TCBT arm, completing four or more of the initial telephone CBT sessions will be deemed as compliant.

Any modifications or departures from randomised treatments, and withdrawal of participants from trial treatment or research follow-up, will be recorded and reported as such.

### Putative mediator variables

Mediator variables include cognitions and behaviours that form part of the cognitive behavioural treatment model and are targets of the therapies.

These include

Cognitive Scale for Functional Bowel Disoders (CG-FBD),^30^ a 31 item scale assessing unhelpful cognitions related to IBS.

Brief Illness Perception Questionnaire for IBS (IPQ),^31^ consisting of an eight point scale to assess participants perception of their illness.

The Belief about Emotions Scale (BES),^33^ a 12-item questionnaire that measures beliefs about the unacceptability of experiencing and expressing negative emotions. These beliefs are likely to have implications for emotion regulation and processing.

The Irritable Bowel Syndrome-Behavioural Responses Questionnaire,^32^ a 26 item scale that measures changes in behaviour specific to managing IBS symptoms.

The “Impoverished Emotional Experience (IEE)” factor of the Emotional Processing Scale,^34^ which is composed of five items, and relates to the labelling and awareness of emotional events that influence the way people process their emotions.

The Positive and Negative Affect Schedule (PANAS),^35^ which measures both positive and negative affect. The current results indicate that positive and negative effect are relatively independent dimensions. Participants will complete only the positive effect subscale because the HADS scale will already measure negative effect.

### GP notes review

Patients’ GP notes will be reviewed at 12 months to assess GP and other consultations in the year prior to entering the study and in the 12 months since entry into the study. Other studies have shown an impact on GP contacts from patient self-management programmes.^16^ ^37^

### Qualitative component

A nested qualitative study will explore patients’ experiences of treatments. The objectives of this study are: to identify factors that facilitate or impede adherence to web-delivered and therapist-delivered CBT; to provide insight into the quantitative results; and to explore social and psychological processes of change. Semistructured interviews will be conducted at 3 and 12 months, with approximately 17–20 participants per arm (ie, 10–12%, sampled purposively to encompass a mix of gender and ages, and a range of baseline symptom severity scores). Interviewers will use topic guides comprising open-ended questions and prompts designed to elicit participants’ accounts of their experiences of IBS in relation to the trial. Interviewing participants from each active arm will enable us to identify factors related to adherence and change processes; including participants from the TAU arm will provide insight into the quantitative results. Interviewing the same participants at 3 and 12 months will allow us greater depth to explore change processes over time and provide the potential to understand better any differences in the quantitative results between 3 and 12 months. Interviews will be transcribed verbatim. Analysis will begin on completion of the first few interviews and will proceed iteratively, thus allowing early insights to be explored more fully in later interviews and topic guides to be modified if necessary. Inductive thematic analysis,^38^ employing supplementary techniques from grounded theory,^39^ will be used to code the data and to identify themes that capture key concepts and processes. To enhance quality: multiple researchers will contribute, to avoid producing idiosyncratic interpretations; a ‘member check’ will be conducted whereby interviewees will be invited to comment on summaries of their interviews; and an audit trail (coding manual and notes) and interviewers’ field notes will be produced. The qualitative results will thus provide insights into the relative merits of each type of CBT and identify delivery issues to attend to in any future widespread implementation.

### Proposed sample size

Sample size was based on the two primary outcomes, IBS-Symptom severity score (IBS-SSS)^23^ and WASAS.^24^ ^40^

A 35 point difference between therapy groups and TAU on IBS SSS at 12 months is regarded as clinically significant (assuming a 15 point placebo response in the TAU arm in the trial).^23^ Assuming a within-group IBS SSS standard deviation (SD) of 76 points (taken from MIBS pilot study^14^), this equates to an effect size of 0.46. To achieve 90% power to detect such an effect or larger using a two-sided independent samples t test at the 2.5% significance level (adjusting for 2 primary outcomes), would require 119 subjects per group. Based on each of 10 therapists delivering therapy to 17 patients within WBCBT and TCBT groups, and an intraclass correlation of 0.02, taken from Baldwin,^41^ this sample size needs to be increased by an inflation factor of 1.32, to take account of therapist effects. We will measure IBS SSS at baseline and assume that baseline values are predictive of post-treatment values (correlation 0.4). Accounting for this in our statistical analysis model allows us to decrease the sample size by a deflation factor of 0.84. Finally, assuming that attrition will be less than 20%, we apply a further inflation factor (factor 1.25) to allow for this. The final sample size requirement is 165 patients per group, or 495 patients in total.

In terms of our second primary outcome (WASAS), this sample size would be sufficient to detect a clinically important difference between the WBCBT (or TCBT) and TAU groups in the WASAS. Specifically, we can assume inflation factors of 1.32 for correlation of outcomes within therapists, and of 1.25 for attrition and a deflation factor of 0.84, for correlation between baseline and follow-up measures. Therefore, a moderate effect size of 0.46 could be found with 90% power at the 2.5% significance level, given 119 participants per group. Assuming a SD of 8.0 (as estimated in a study of CBT for IBS^6^), this would equate to a clinically meaningful treatment difference of 3.7 points on this scale. This is less than the difference of 5.4 points in change of means that was found in a trial of a CBT-based self-management intervention for IBS.^16^

### Statistical analysis

The Statistical analysis plan has been approved by the trial steering committee. The aim is to evaluate effectiveness, and all analyses will follow the intention-to-treat principle. Group differences on the primary IBS-SSS outcome will be assessed using a mixed linear regression model for repeated measurements. In this model, IBS-SSS at post-treatment time points (3, 6 and 12 months) will feature as the dependent variable. Explanatory variables will be baseline IBS-SSS, treatment group, IBS symptoms type, stratifier (centre) and time and time by treatment interaction terms to allow for different group differences at the various assessment time points. (The assessment time point of primary interest is 12 months. The modelling provides the treatment effect estimates at the 12 month time point as well as for further post-treatment secondary time points). Correlation between repeated measures of the same participant or between participants, or due to sharing the same therapist, will be allowed for by including subject-varying random intercepts as well as therapist-varying random intercepts for TCBT and WBCBT groups in the mixed models. Mixed models account for missing outcome data under the missing at random assumption. The effect of departures from this assumption will be checked using sensitivity analyses.^42^ WASAS scores will be analysed using mixed models in a manner similar to the analysis of IBS-SSS. Secondary outcomes, including Subjects’ Global assessment of relief (SGA), EQ-5D, Enablement, HADS, Brief Illness perception Questionnaire (IPQ), Cognitive Scale for Functional Bowel disorders, The Belief about Emotions Scale (BES), The “Impoverished Emotional Experience (IEE)” factor of the Emotional Processing Scale, The Positive and Negative Affect Schedule (PANAS), adverse events (AEs) and healthcare utilisation, are important to measure the wider IBS effects and will be analysed similarly (as appropriate for continuous or dichotomous outcomes). A complier average causal treatment effect will be estimated using instrumental variable methods to assess efficacy if there is appreciable lack of compliance.^43^

### Economic evaluation

We will measure costs and assess cost-effectiveness from both a health service and a societal perspective. To calculate the cost of TCBT, the number of sessions with therapists will be recorded and combined with the unit cost of therapist time. The latter will be calculated using information on the salary band of therapists, with additional costs representing capital, overheads, training and qualifications.^44^ We will ask therapists to estimate how much time during a typical working week is spent in telephone contact with patients, and combine this with the total cost and total hours worked per week, in order to produce a cost per hour of direct patient contact time. For WBCBT, the number of times therapist support is provided will be recorded and costed in a similar way. The WBCBT development costs will be estimated and apportioned over those using the intervention. Other service use will be measured with a service receipt schedule at baseline (going back 6 months) and at each follow-up (with measurement covering the whole period since the prior interview). The schedule will be based on other questionnaires used in similar research.^29^ Services will include primary and secondary healthcare, and medication. Service costs will be generated by combining these data with appropriate unit cost information (eg, NHS Reference Costs,^44^ and the British National Formulary) and these costs added to the intervention costs in order to generate total health costs per person.

Societal costs will be calculated by including family care costs and lost production. Family care costs will be recorded by asking patients to state how much time per week family members (and friends) spent providing support in specific areas *because of the IBS*. This time will be combined with average wage rates. Lost days and hours from work will be recorded on the schedule and combined with average wage rates to generate lost production costs. Cost comparisons between the three groups will be made at 3, 6 and 12 months, and over the entire follow-up period, in both cases, controlling for baseline costs. Cost data are usually skewed and cost comparisons will use a bootstrapped regression model to generate appropriate 95% CIs around the cost differences.

Cost-effectiveness will be assessed (from health and societal perspectives) by combining the cost data with the change score on the IBS-SSS, WASAS and QALYs. The latter will be generated from the EQ-5D combined with UK-specific tariffs. Area under the curve methods, controlling for baseline utility, will be used to calculate the number of QALYs accrued over the follow-up period. If outcomes are better for one group compared to another and costs are lower, then it will be defined as being ‘dominant’. If outcomes are better and costs are higher, an incremental cost-effectiveness ratio will be generated to indicate the extra cost incurred to achieve an extra point reduction in symptoms or extra QALY. Cost-effectiveness planes will be produced, using 1000 cost and outcome differences (from bootstrapped regression models) for each 2-way comparison, to explore the uncertainty around the results. Cost-effectiveness acceptability curves will also be produced using bootstrapped regression models with net benefit values as the dependent variables. The net benefit approach requires an assumption about the value placed on a unit improvement in outcome. For QALYs, a range from £0 to £60 000 will be used, thus including the threshold thought to influence NICE decisions. For the IBS-SSS and WASAS, there are no accepted thresholds, so a range will be chosen such that the points at which one intervention has a 60%, 70%, 80% and 90% likelihood of being the most cost-effective option can be identified.

Sensitivity analyses will be conducted by changing the intervention costs upwards and downwards by 50%, using minimum wages to value lost production, family care and travel time, and by also using the replacement cost approach to value family care with the cost of a homecare worker used a shadow price.

Modelling beyond the trial period and making comparisons with other interventions is not in the scope of this project.

## Ethics and dissemination

### Ethical issues

The trial will be conducted in full accordance with current guidelines for ethical research conduct. The study will be performed subject to Research Ethics Committee (REC) approval, including any provisions of Site Specific Assessment (SSA), and local Research and Development (R&D) approval.

The potential benefit to participants from the interventions in this study is a greater understanding of their IBS, an improved ability to manage their condition and possibly reduced symptom severity or impact on their life from their IBS. This may lead to societal benefits such as a reduction in work days lost and reduced use of NHS resources. The risks of undertaking CBT are minimal; undertaking the sessions will require a time commitment on behalf of the participants, and focusing on their IBS symptoms could temporarily worsen the symptoms in the short term. The CBT is provided alongside usual care so the participants will still have access to all usual NHS services. Participants will be fully informed of the trial procedures before entering the study via a Patient Information Sheet, and any questions will be answered by the research team prior to signing the on-line consent form.

#### Fair access to the study

Participants need to have web access, which could exclude some people who would otherwise like to take part. However, three quarters of households have web access, this figure is rapidly increasing, and those without home access can use public computers (eg, local library).

Participants aged over 60 years are required to have undergone a consultant review to exclude other serious causes of their bowel symptoms in the last 2 years, because colorectal cancer is more common in those aged over 60 years and guidelines recommend that changes in bowel habit in this group require hospital tests beyond the scope of this trial.

To maximise recruitment and to ensure that motivated patients are not excluded from treatments that may help, participants in the TAU-alone group will be given access to the Regul8 website at the end of the trial.

### Dissemination

The results of this study will be communicated to participants at study end and disseminated via peer reviewed publications and conference presentations. The results will enable clinicians, patients and health service planners to make informed decisions regarding the management of IBS with CBT.

### Service users

IBS patients and the IBS network, a patient self-help group, have been involved in providing feedback for the design of the MIBS^14^ trial (in which the Regul8 website used in this study was developed and piloted). Patients were substantially involved in the website design with service users, working through each on-line module during development and providing ‘Think Aloud’ feedback to inform the design. Participants from the MIBS trial have also provided input and feedback on the proposals for this research. Two participants are now PPI representatives for this study, providing ongoing input (both informal feedback and participating in Trial Steering Committee (TSC) and research meetings) to ensure it addresses issues relevant to users.

### Research governance

This study will be conducted in accordance with the International Conference for Harmonisation of Good Clinical Practice (ICH GCP) guidelines, and the Research Governance Framework for Health and Social Care. The University of Southampton is the Sponsor for this study.

A Trial Steering Committee (TSC) will oversee the trial procedures and ensure good conduct of the study; they will meet at least annually. Observers from the HTA will be invited to all TSC meetings.

A Data Monitoring and Ethics Committee (DMEC) will oversee the trial data and ethics, with an independent chair and at least two independent members, and a Patient and Public Involvement Representative, along with the lead investigator (HE), with the support of the TSC. They will meet at least annually.

Regular updates and meetings will ensure good communication. The collaborators will hold meetings at least four times a year. The research assistant will circulate a monthly update to review progress relative to the project plan, highlighting any issues that need to be addressed. Each team member will consult the other team members immediately by email and/or phone on any issues that arise.

### Monitoring and audit

The study will be monitored and audited in accordance with Southampton University procedures. All trial related documents will be made available on request for monitoring and audit by the University of Southampton, the relevant REC and other licencing bodies.

### Safety

#### Adverse events

AE are any clinical change, disease or disorder experienced by the participant during their participation in the trial, whether or not considered related to the use of treatments being studied in the trial.

#### Serious adverse events

An AE is defined as serious (an SAE) if it results in one of the following outcomes

- A life-threatening AE
- In-patient hospitalisation
- A disability/incapacity
- A congenital anomaly/birth defect in the offspring of a subject
- Other medical events requiring intervention to prevent one of the above outcomes.

#### Serious adverse reactions

A serious adverse reaction (SAR) can be defined as: A SAE considered to be a reaction to one of the supplementary therapies.

#### Reporting serious adverse events and reactions (SAEs and SARs)

On completion of an SAE, the chief investigator will assess whether the SAE is a SAR or a (SUSAR). A SUSAR is any adverse reaction that is classed as serious and is suspected to be caused by the intervention, and is not expected. If the SAE is classified as a SUSAR, the trial team will report the SUSAR to the EC. For a SUSAR that is fatal or life-threatening, the team, on behalf of the sponsor, has 7 days to report the SUSAR to the EC. For a SUSAR that is not fatal or life-threatening, the team has 15 days to report. The SUSAR is recorded in the participant’s medical notes and the participant will be followed up.

#### Follow-up after AEs

After a SAE or SAR, a decision will be made by the trial team, after advice from the relevant authorities and the participant's GP, as to whether the participant should be withdrawn from either their randomised treatment or from the trial. Arrangements will be made by the trial team for further assessment and management as agreed with the relevant authorities, GP and participant. The investigator will provide the trial team with a 1-month follow-up report on all SAEs and SARs. Further monthly reports should be provided in the absence of resolution. These reports will be communicated to the TSC, DMEC and MREC, and to the local R&D office. Blank Adverse Event Forms will be distributed to sites that are recruiting, and therapists and patients will be prompted to self-report SAEs in the follow-up questionnaires.

#### AEs that do not require reporting

Expected AEs include planned/elective hospitalisations, and these will not be collected as SAEs.

### Stopping rules

The trial may be prematurely discontinued by the Sponsor or Chief Investigator on the basis of new safety information or for other reasons given by the Data Monitoring & EC, Trial Steering Committee, Regulatory Authority or EC concerned.

The trial may also be prematurely discontinued due to lack of recruitment or on advice from a Trial Steering Committee (if applicable), who will advise on whether to continue or discontinue the study and make a recommendation to the sponsor. If the study is prematurely discontinued, active participants will be informed and no further participant data will be collected.

#### Data protection and anonymity

Data will be collected and retained in accordance with the Data Protection Act 1998.

The Data Protection policy of the School of Medicine, Southampton University, will be complied with.

GP participants will be identified from Health Authority lists (these are available in the public domain) and via the CRN.

The responses to questionnaires will be stored in an anonymised form on a password protected university or CTU server. Any anonymised paper questionnaires will be stored in a locked filing cabinet at Primary Medical Care—University of Southampton, or at King's College London.

#### Storage of records

Study documents (paper and electronic) will be retained in a secure location during and after the trial has finished. All source documents will be retained for a period of 10 years following the end of the study.

## Conclusion

This paper outlines the protocol for the ACTIB study. This study has significant strengths: to date, ACTIB, when completed, will be the largest trial worldwide to address the clinical-effectiveness and cost-effectiveness of CBT for IBS and has the advantage of comparing a low intensity web-based CBT (WBCBT) with a higher intensity telephone delivered CBT (TCBT). Additionally, ACTIB will recruit from both primary and secondary care, inviting a broad range of patients with refractory IBS from specialist as well as community settings. This will aid generalisability of the findings.

The limitations of this study are that due to the online nature of the Low intensity CBT arm, patients without internet access will be unable to participate. However, internet access in the UK is currently over 75% and those without home access can use public computers (eg, local library). Also, participants aged over 60 years are required to have undergone a consultant review to exclude other serious causes of their bowel symptoms in the past 2 years, because colorectal cancer is more common in the those aged over 60 years, and guidelines recommend that changes in bowel habit in this group require hospital tests beyond the scope of this trial.
